# *P*53 gene codon 72 polymorphism in patients with oral squamous cell carcinoma in the population of northern Iran

**DOI:** 10.4317/medoral.19794

**Published:** 2014-06-01

**Authors:** Mahmud Sina, Mehrdad Pedram, Morteza Ghojazadeh, Ahmad Kochaki, Amirala Aghbali

**Affiliations:** 1 Department of Oral and Maxillofacial Pathology, Faculty of Dentistry, Tabriz University of Medical Sciences (TBZMED), Tabriz, Iran; 2 Department of Genetics and Molecular Medicine, School of Medicine, Zanjan University of Medical Sciences (ZUMS), Zanjan, Iran; 3 Liver and Gastrointestinal Disease Center, Tabriz University of Medical Sciences (TBZMED), Tabriz, Iran

## Abstract

Objectives: Squamous cell carcinoma is the most common cancer of the oral cavity, and several etiologic factors are involved in its development. Single nucleotide polymorphism (SNP) of the *P*53 gene codon 72 (*P*53c72) changes the structure of the protein and affects its activity. The prevalence of *P*53c72 different genotypes, which seems to vary with race and geographic location, has shown a strong correlation with many types of human cancers. The aim of this study was to investigate the correlation between *P*53c72 polymorphism and risk of oral squamous cell carcinoma (OSCC) in the heavily populated Gilan Province in northern Iran. Design of Study: This case-control study was done on 55 paraffin-embedded samples from OSCC patients and 100 samples of non-dysplastic oral cavity lesions. The *P*53c72 genotypes were determined using the ARMS-PCR method. SPSS-15 software was used for statistical analysis. Results: There were no significant statistical differences found between the prevalence of different *P*53c72 genotypes in the OSCC group vs. the control. However, the Pro/Pro genotype in OSCC samples showed a strong correlation with age, as 70% of such patients were below 50 years old. Interestingly, a large portion (40%) of the patients with the Pro/Pro genotype had the tumor in the lip area. Conclusions: Although *P*53c72 polymorphism does not appear to be a predisposing factor for OSCC in the population of Northern Iran, the Pro/Pro genotype could be considered as a risk factor for OSCC in adults below 50 years old and the anatomical location of the tumor.

** Key words:**OSCC, P53 codon 72 polymorphism, northern Iran.

## Introduction

Cancer remains a major problem among human societies. Oral squamous cell carcinomas (OSCC) are among the most common cancers in both sexes worldwide (http://www-dep.iarc.fr/). Epidemiological studies indicate that the cause is multi-factorial and includes nutrition, use of tobacco and/or alcohol, viral infections, genetic factors, and UV exposure. There is also some evidence for involvement of some genetic predisposing factors for OSCC. Such genetic changes could occur in oncogenes, tumor suppressors, and growth regulator genes (1).

The *P*53 protein is encoded by a key tumor suppressor gene (*P*53) with 11 exons and 10 introns located on the short arm of chromosome 17. *P*53 regulation plays an important role in the control of cell cycle and DNA damage response. In fact, *P*53 mutations are a common genetic event in most cancers, and many such mutations increase cell proliferation, hamper apoptosis, and often lead to genetic instability (2,3).

It has been reported that in addition to mutations, genetic polymorphisms could also have an influence on the *P*53 performance. In particular, there is an interest in *P*53 codon 72 (*P*53c72) single nucleotide polymorphism (SNP) that could result in either arginine (Arg) or proline (Pro) alleles and create 3 different genotypes: Arg/Arg, Arg/Pro, and Pro/ Pro (4). There have been reports showing possible involvement of *P*53c72 polymorphism in individuals’ susceptibility to cancers of mouth (5), breast (6), colorectal (7), lung (8), and bladder (9). In fact, the *P*53c72 polymorphism does seem to have a significant impact on the *P*53 protein function: the presence of Arg at this position, compared to Pro, has been shown to result in a greater ability to induce apoptosis in vitro. These two different alleles also differ in terms of protein structure, transcriptional activity, and carcinogenesis (10).

The impact of the *P*53c72 polymorphism appears to depend on geographic distributions and race. Although OSCC is common in Iran, there have been only a few studies, with limited scopes, conducted on it in some limited parts of the country (11-13). Therefore, additional research could provide some useful information and insight on OSCC etiology among the Iranian population. The main aim of this study was to assess the rate of different *P*53c72 genotypes in OSCC samples from the city of Rasht, the center of the heavily-populated Gilan province that is located in the north of Iran. Many of the residents in this area belong to the Gilak ethnic group and a good number of them are farmers.

## Material and Methods

2.1. Samples and Selection Criteria

In this case-control study, 55 paraffin-embedded samples from patients with OSCC, as the case group, and 100 samples from patients with non-dysplastic lesions of the oral cavity, as the control group, were obtained from the Central Laboratory of the Facial Lesions in the Province of Gilan. The case group consisted of all patients with OSCC registered from 2005 to 2011 in the area of study. All OSCC diagnoses were reviewed and approved by two independent Oral and Maxillofacial pathologists.

2.2. *P*53 Genotyping

Genomic DNA extraction was done by using QiaAmp DNA mini kit (QIAGEN) following the manufacturer’s instruction. To remove RNA contamination, RNAse A was used during the extraction process. In order to control the quality and concentration of the extracted DNA samples, each sample was analyzed by a nano-drop spectrophotometer at wavelengths of 260 and 280 nm.

The ARMS-PCR process using specific primers was done with the following steps:

Each PCR was performed by using 200-250 ng of gDNA, 10 mM Tris-Hcl, 50 mM KCl, 2.5 mM MgCl2, one unit of Taq DNA polymerase enzyme, 200 mM of each of the nucleotides (dGTP, dTTP, dCTP, and dATP), and 50 pico moles of each pair of specific primers for amplification of either proline or arginine ( Table 1).

**Table 1:**
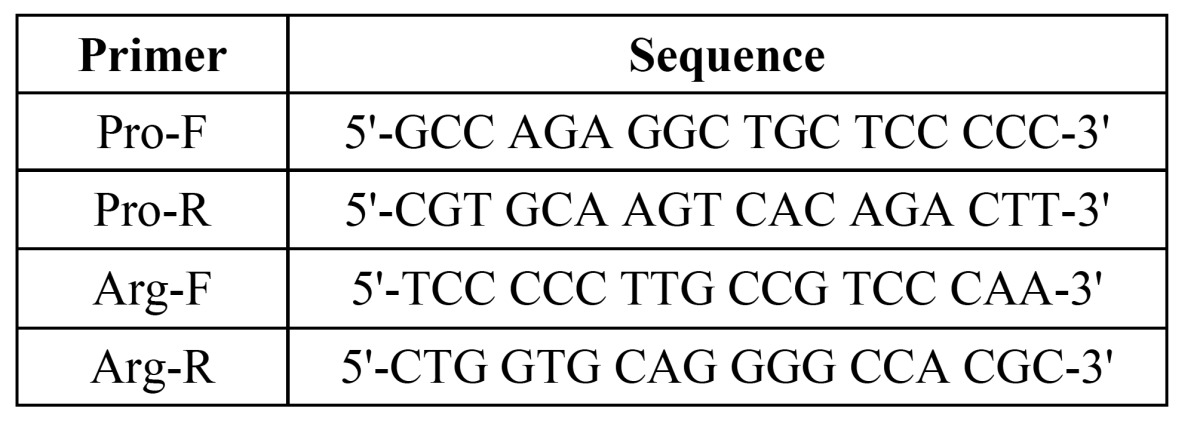
Sequences of the primers used for ARMS-PCR.

All reactions were done with Corbett: Rotor-gene 6000. The PCR program for amplification of the Arg SNP was 5 minutes at 94°C, followed by 35 cycles of 45 sec at 94°C, 45 sec at 61°C, and one min at 72°C, followed by a 10 min cycle at a of 72°C. For the Pro SNP amplification, the program ran as 5 minutes at 94°C, followed by 35 cycles of 45 sec at a 92°C, 45 sec at 55°C, and one minute at 72°C, followed by a 10 min cycle at 72°C.

In order to analyze the reaction products, 20 µl of each reaction was mixed with 4 µl of the loading buffer, run on a 2% agarose gel in 0.5 X TBE buffer, stained with ethidium bromide, and visualized on a UV transluminator.

2.3. Statistical Analysis

The data were analyzed using the SPSS version 15 software and applying the ChiSquare or Fisher exact tests. *P*-value less than 0.05% was considered significant.

## Results

A total of 55 OSCC cases including 29 males and 26 females with a mean age of 65.87 ± 14.72 years (ranging from 27 to 90 years) were studied. Comparisons of the incidence of OSCC are shown on  Table 2. The majority (25.45%) of the tumors were on the lips, followed closely by tongue (21.81%), buccal mucosa (BM; 20%), and gums (18.18%). The least affected areas were the palate and floor of the moth (FOM) with only 2 and 5 incidences out of the total of 55, respectively. As the control group, one hundred individuals, including 50 males and 50 females, with a mean age of 62.69 ± 12.99 years and non-dysplastic lesions were included in the study. In the control group, the lesions were only due to the stimulation of the oral cavity including fibrous lesions (stimulative fibroma) and pyogenic granuloma, in which only the connective tissue had shown reactive changes with no neoplastic activity. Statistical analysis showed no significant differences regarding either the male and female proportions or the average age of the patients between the test and control groups.

**Table 2:**
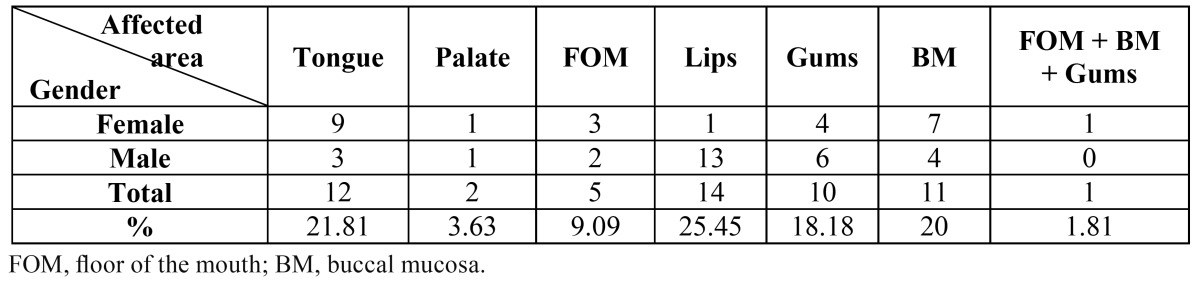
Demographic details of OSCC patients and tumor sites.

**Figure 1:**
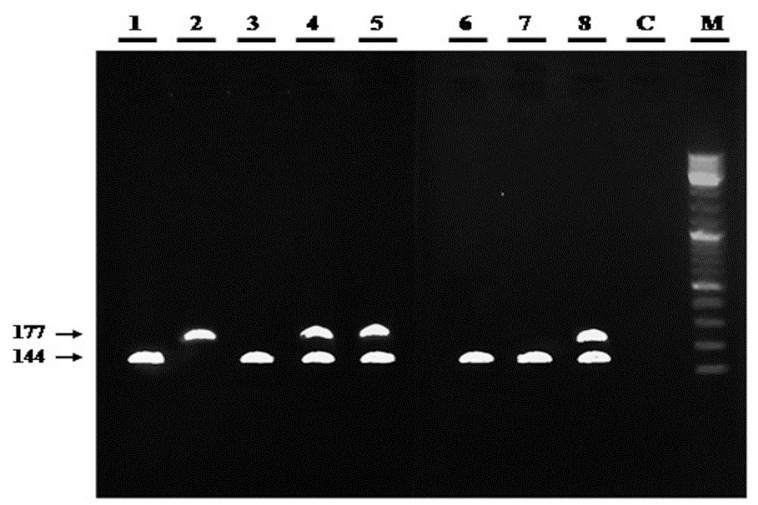
Examples of P53 genotypes amplified by ARMS-PCR and run on an agarose gel. Lanes: M, DNA ladder; C, negative (no-template) PCR control; 1-8, test PCR samples (genotypes: Arg/Arg, lanes 1, 3, 6, and 7; Pro/Pro, lane 2; Arg/Pro, lanes 4, 5, and 8).

Analysis of the frequency of the P53c72 polymorphism in the test group shows that out of the total of 55 OSCC patients, 45.5% of them had the Arg /Pro genotype, followed by Arg /Arg (36.4%) and Pro/Pro (18.2%). As shown in figure 1, in samples with the Pro/Pro genotype, a dedicated band of 177 bp was observed, in contrast to a 141-bp band for the Arg/Arg genotype. As expected, some samples with a heterozygote genotype contain both Arg and Pro bands. A detailed analysis of the distribution of P53c72 alleles and genotypes for OSCC cases vs. the controls is presented on  Table 3.

**Table 3:**
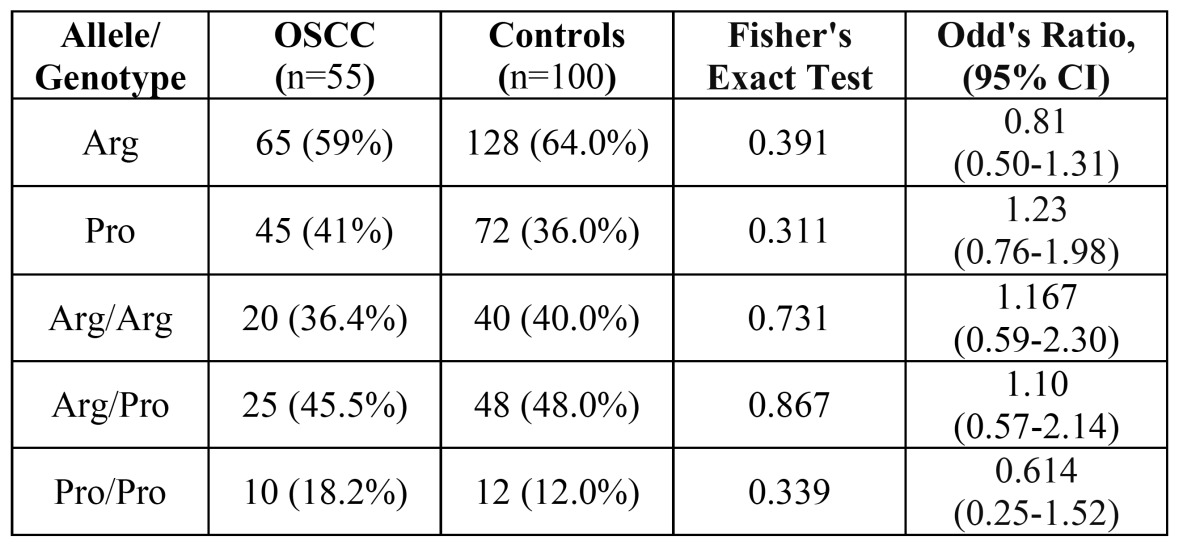
Distribution of the P53 codon 72 alleles and genotypes in OSCC samples and controls.

As it can be seen from the results, the frequency of the Arg72 allele is 5% higher among the OSCC patients compared with the controls. There is also a 6.2% higher Pro/Pro genotype among the OSCC samples. However, these values are not statistically significant. As for the distribution of tumor sites, the highest frequencies of Arg/Pro and Arg/Arg genotypes were observed in the tongue (32%) and lips (35%), respectively ( Table 4).

**Table 4:**
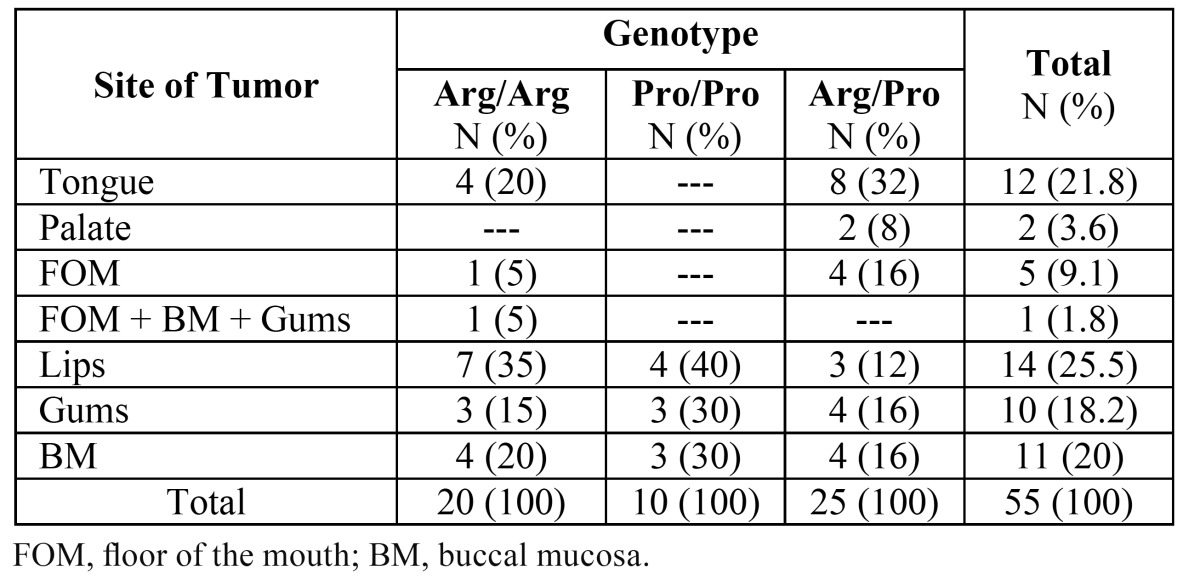
Distribution of the involved area and correlation with P53c72 genotypes in OSCC patients.

Interestingly, when the patients are divided into two different age groups of either under 50 or equal and over 50 years, we notice that the Arg/Arg and Arg/Pro genotypes are highly prevalent among those above 50-years-old ( Table 5). In fact, the vast majority (93.3%) of the OSCC patients above 50 had either the Arg/Arg (19; 42.2%) or Arg/Pro (23; 51.5%) genotypes. As for the Pro/Pro genotype, however, about 70% of the patients had less than 50 years.

**Table 5:**
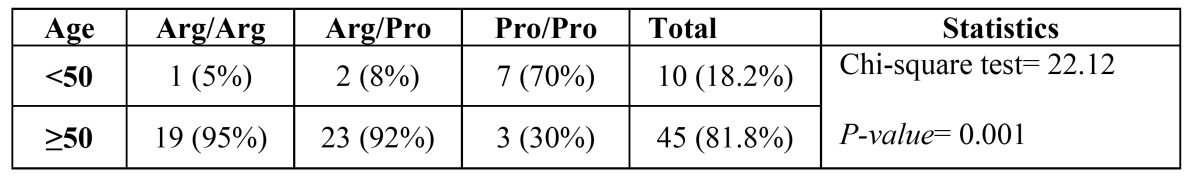
The frequency of the 3 different P53c72 genotypes in patients either below or equal and above 50 years old.

## Discussion

Mutations of *P*53, a key tumor suppressor gene that plays central roles in response to DNA damage, control of the cell cycle, and regulation of apoptosis, have been linked with many humans cancers (14). Depending on the geographical area, the rate of different mutations can vary from 30 to 70 percent (15). About 90% of these mutations lead to the synthesis of a stable but nonfunctional **P53 protein accumulating in the nuclei of tumor cells (16).

Occurrence of a Single Nucleotide Polymorphism (SNP) at codon 72 in exon 4 of the P53 leads to the presence of either Arg or Pro alleles, which in turn could result in 3 different genotypes: Arg/Arg, Arg/Pro, and Pro/Pro. Although the substitution of the Arg codon with Pro, or vice versa, affects the structure of the *P*53 protein, the mechanism by which this might affect *P*53 function remains unclear. In comparison to the Pro72 allele, Arg72 seems to induce apoptosis by increasing *P*53 localization to mitochondria (17).

The involvement/effect of *P*53c72 polymorphism in various cancers has been controversial. For example, Storey and colleagues have shown that excessive expression of homozygous Arg72 *P*53 protein can increase susceptibility of cervical cancer associated with HPV for up to seven fold (4). By contrast, Liu and colleagues have reported that the Pro72 *P*53 allele is associated with increased risk for lung adenocarcinoma and SCC (18). In another study by Twu and colleagues, it has been reported that the heterozygote Arg/Pro genotype is associated with an increased risk of hypopharyngeal SCC (19). While these findings have been confirmed by some other studies (20-23), they have also been contested by some investigators (24,25).

There is limited information on the impact of *P*53c72 polymorphism on OSCC. In a study carried out in Taiwan, Bau and colleagues reported that the Arg/Arg genotype seems to increase the risk of oral carcinoma by 2.7 fold (26). By contrast, we found no significant association between *P*53c72 polymorphism/genotypes and OSCC among the population of the Gilan Province in northern Iran. Our observation is similar to the results obtained in a study by Shen and colleagues on head and neck SCC (1), as well as Katiyar *et al*. on HPV-associated oral cancer in India (5). It is of course possible that such variations might be due to a variety of factors including racial and geographic differences or predisposing factors such as Alcohol consumption, tobacco use, and life style and risk of HPV viruses.

The mean age of patients with OSCC in the present study was 65.87 ± 14.72 years. On average, female patients were about 6 years older than males. This was similar to the studies done by Sankarnarayanan *et al*. (27) and Jovanovic *et al*. (28) in Netherlands and India, but different from Otho *et al*. in Nigeria (29), in which the mean age of the male OSCC patients was more than females.

Interestingly, among the samples studied here, the lip was the most common anatomical site of tumor involvement (25.45%) following by tongue (21.81%) and buccal mucosa (20%). This is in line with a number of studies, in which either lip (24,25,26,27) or tongue (11,28,30,31) was reported as being the most common tumor site.

An important observation made in our study is that the frequency of OSCC patients under 50 years with the Prp/Pro genotype was 70%, which is statistically significant (*p*=0.001). Significant portions of the OSCC patients with the Arg/Pro and Arg/Arg genotypes had the lesions on their lips and tongues, which can suggest an increased risk of OSCC tumor site with certain genotypes. However, one cannot draw any firm conclusions without investigating a larger sample size. Moreover, additional studies are required to investigate the role of various environmental and ethnic factors in order to determine the role of *P*53 codon 72 polymorphisms in OSCC.
